# Ontorat: automatic generation of new ontology terms, annotations, and axioms based on ontology design patterns

**DOI:** 10.1186/2041-1480-6-4

**Published:** 2015-01-09

**Authors:** Zuoshuang Xiang, Jie Zheng, Yu Lin, Yongqun He

**Affiliations:** University of Michigan, Ann Arbor, MI USA; University of Pennsylvania, Philadelphia, PA USA

**Keywords:** Ontorat, Ontology design pattern, ODP, Quick term templates, QTT, Ontology development

## Abstract

**Background:**

It is time-consuming to build an ontology with many terms and axioms. Thus it is desired to automate the process of ontology development. Ontology Design Patterns (ODPs) provide a reusable solution to solve a recurrent modeling problem in the context of ontology engineering. Because ontology terms often follow specific ODPs, the Ontology for Biomedical Investigations (OBI) developers proposed a Quick Term Templates (QTTs) process targeted at generating new ontology classes following the same pattern, using term templates in a spreadsheet format.

**Results:**

Inspired by the ODPs and QTTs, the Ontorat web application is developed to automatically generate new ontology terms, annotations of terms, and logical axioms based on a specific ODP(s). The inputs of an Ontorat execution include axiom expression settings, an input data file, ID generation settings, and a target ontology (optional). The axiom expression settings can be saved as a predesigned Ontorat setting format text file for reuse. The input data file is generated based on a template file created by a specific ODP (text or Excel format). Ontorat is an efficient tool for ontology expansion. Different use cases are described. For example, Ontorat was applied to automatically generate over 1,000 Japan RIKEN cell line cell terms with both logical axioms and rich annotation axioms in the Cell Line Ontology (CLO). Approximately 800 licensed animal vaccines were represented and annotated in the Vaccine Ontology (VO) by Ontorat. The OBI team used Ontorat to add assay and device terms required by ENCODE project. Ontorat was also used to add missing annotations to all existing Biobank specific terms in the Biobank Ontology. A collection of ODPs and templates with examples are provided on the Ontorat website and can be reused to facilitate ontology development.

**Conclusions:**

With ever increasing ontology development and applications, Ontorat provides a timely platform for generating and annotating a large number of ontology terms by following design patterns.

Availability: http://ontorat.hegroup.org/

## Background

The Web Ontology Language (OWL) has been widely used for ontology development. However, ontology development and updating in OWL format is often time consuming and requires specialized knowledge of ontology tools as well as specific scientific domains. Ways to improve the process of ontology development are desirable. It is frequently observed that a large number of new ontology terms and term annotations follow the same design patterns of logical definitions and axioms. An ontology term refers to a term with a Uniform Resource Identifier (URI) in the ontology. Even with the help of the Protégé-OWL editor (http://protege.stanford.edu/), manual adding and editing of these terms and annotations is labor-intensive and time-consuming. To make the ontology development more efficient, it is possible to develop tools to automate the process of adding the ontology contents with repetitive design patterns.

An OWL format ontology includes a set of axioms that provides explicit logical assertions about three types of entities - classes, individuals and properties. As with software design patterns for software engineering, an Ontology Design Pattern (ODP) represents a reusable solution to solve a recurrent modeling problem in the context of ontology engineering. ODPs can be applied to support ontology rational design and development, improve ontology quality and reuse, disambiguate relations, provide scalable representations of entities, and make ontologies more maintainable and understandable [1–5]. The web portal of ODPs (http://ontologytdesignpatterns.org) has collected many ODPs in different fields [6]. ODPs have also been studied in biological and biomedical fields [1–4, 7, 8]. ODPs can be represented using ontological axioms or graphic diagrams.

Since many ontology terms (*e.g.*, assays, vaccines) follow the same design patterns, it is possible to apply specific ODPs in new ontology term generations to support ontology enrichment and expansion. To support quick generation of new ontology classes, the developers of the Ontology for Biomedical Investigations (OBI) [9] proposed the usage of a Quick Term Template (QTT), which is a spreadsheet template for populating terms to define specific ontology classes [10]. The populated template spreadsheet can then be converted into an OWL file with newly generated ontology classes. The generation of QTT templates relies on repeatable patterns of to-be-generated ontology classes [10]. The conversion of an input file generated using a QTT template to an OWL output document could be implemented using MappingMaster, a plugin program in the Protégé-OWL editor [11, 12]. The MappingMaster plugin works in Protégé-OWL editor version 3.4 that only supports OWL 1. However, the tool does not function in Protege 4.0 or higher versions that support OWL 2.0 and have become the main choices of ontology developers.

Inspired by the ODP theories and OBI project QTT operation, we developed Ontorat (http://ontorat.hegroup.org/), a web application with the aim to automatically generate a large number of new ontology classes or add additional axioms (*e.g.* annotations) to existing classes for a specific target ontology. Ontorat offers a web-based platform for writing up ontology axiom expressions with variables. Based on the axiom settings and a user-provided input data file populated on a QTT-like template, Ontorat is able to generate an OWL format output file, which can be imported into a target ontology to enrich and expand the ontology. Ontorat was first presented in the ICBO-2012 conference as a software demo [13]. The tool has been much improved during the past two years, including bug fixes, web user interface improvements, and new feature additions. Ontorat has been used in enriching several widely-used ontologies including the Vaccine Ontology (VO) [14, 15], the Ontology for Biomedical Investigations (OBI) [9], and the Cell Line Ontology (CLO) [16]. To allow users to better understand and use the tool, we provide systematic descriptions and use case examples of the Ontorat in this paper.

## Overall design

**Figure 1:**
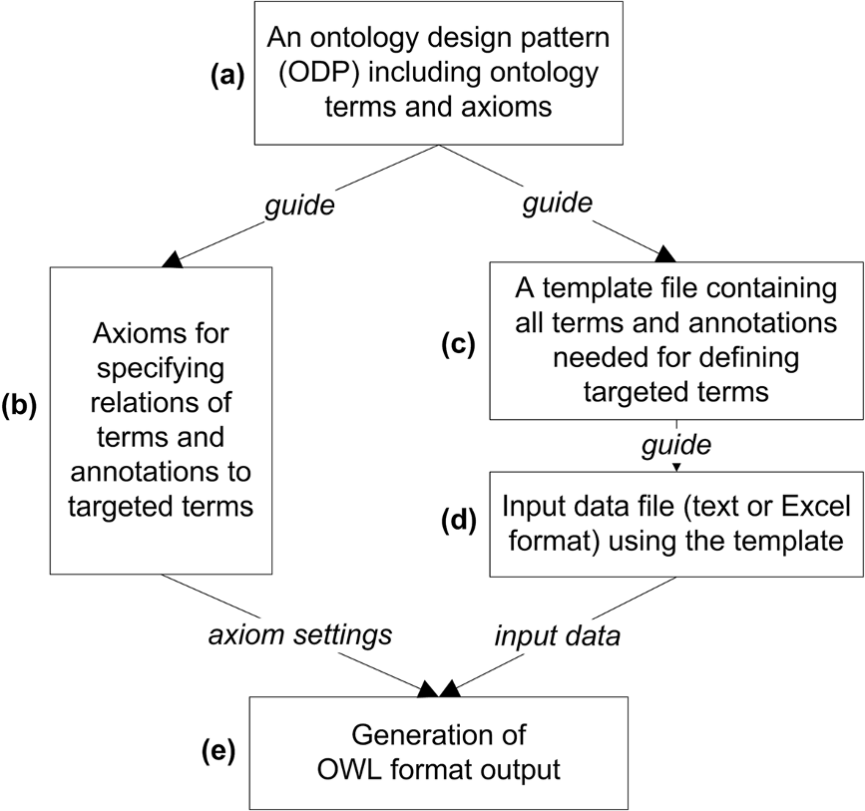
**The strategy of applying ODPs into ontology term and annotation generation.** An ODP is used to guide the generation of axiom settings and a template file (text or Excel format). The template file is populated with specific contents to create an input data file. Based on the axiom assertions and input data file, an OWL output can be generated by a software program to expand a targeted ontology.

Based on the ODP concept and the Quick Term Templates (QTT) procedure, we developed an overall strategy of applying these mechanisms to ontology expansion (Figure 1). First, an ODP that covers a set of terms and their relations needs to be identified (Figure 1a). Formal axioms that assert logical relations among ontology terms and annotations of these terms will then be specified based on the ODP (Figure 1b). The ODP will guide the generation of a tab-delimited text or Excel template file which would contain all terms and annotations needed to define targeted terms (Figure 1c). This template file will then be used to populate specific contents (Figure 1d). By combining the axiom settings and the input data file, an OWL format output can be generated (Figure 1e).

**Figure 2:**
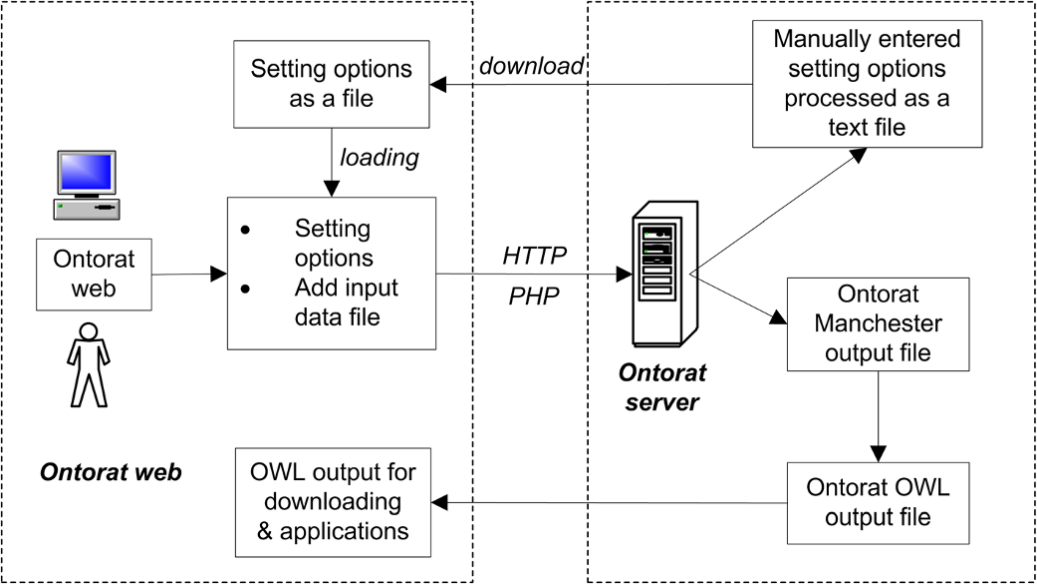
**Ontorat software overall design and workflow.** See the text for description.

We have developed the web-based Ontorat tool that implements the ontology enrichment strategy shown in Figure 1. Figure 2 lays out the Ontorat design and workflow pipeline. Specifically, on the Ontorat web page, a user enters setting options and uploads the input data file via the Ontorat web input form. The input data file is generated by populating a predesigned template file guided by the ODP as mentioned above. After accepting the input data file and setting options from the user, the web server (via a PHP script) will be able to execute two operations: 1) generation of new ontology classes with logical axioms and annotations, or 2) addition of new axioms to existing ontology terms. The Ontorat server will process the user’s requests and generate either an Ontorat settings file or an OWL output file. The Ontorat settings file can be stored and reused later. For the OWL output generation, a Manchester syntax file will be generated first and then transferred to OWL format (Figure 2).

## Implementation

### Sever setup

The Ontorat server is a single HP server running the Red Hat Linux operating system (Red Hat Enterprise Linux 6). The Selinux program is enabled to improve the security and stability of the server. The open source Apache HTTP Server is installed as the HTTP application server. PHP is used as the programming language in the web application server. OWL API is used for OWL format data operations.

**Figure 3:**
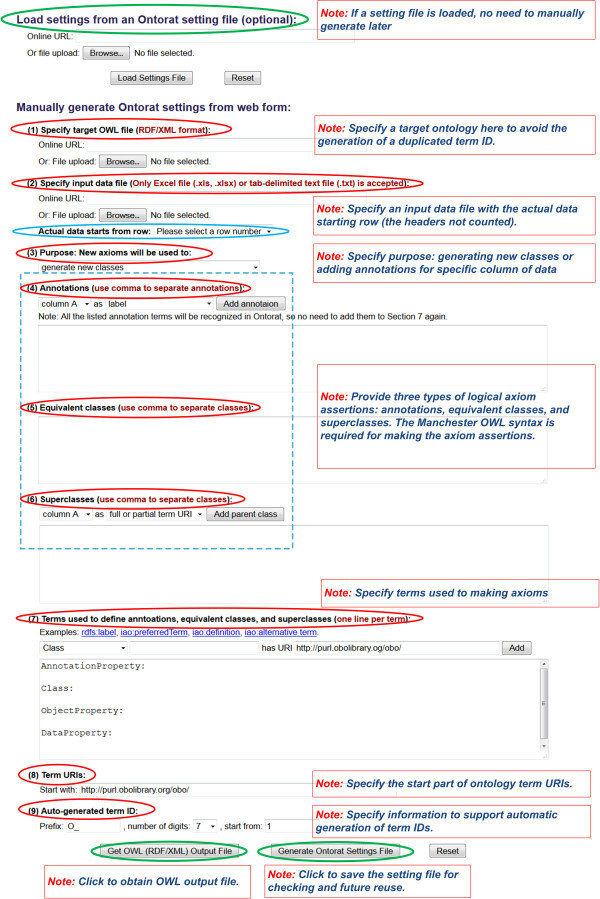
**The Ontorat web interface with explanation.** The balloons represent components of the Ontorat web form for users to provide or click. It is noted that some components are optional. The text notes inside boxes are the explanation notes for specific Ontorat components in the web form.

Ontorat (http://ontorat.hegroup.org) provides a user-friendly web form for data input (Figure 3).

### Ontorat inputs

As guided by the general strategy shown in Figure 1, an Ontorat execution requires two types of required inputs:

•Input data file, Figure 3 (2):
  An Ontorat template file is usually generated first based on the ODP including all term and annotation types needed for defining a target term and then filled up with specific terms and annotations for each type. The file can be provided in an Excel or tab-delimited text format.Axiom settings, Figure 3 (4)-(6):
- The axioms are represented using Manchester OWL Syntax [17] in Ontorat. The axiom settings can be added one by one via the Ontorat web form or uploaded from an Ontorat setting text file in an Ontorat-specific setting file format. Ontorat can also generate the setting file based on the setting inputs via the Ontorat web form.
- Three types of axiom assertions are allowed in Ontorat:

- Annotations. The annotations associate information with an ontology class. Each annotation includes an annotation property with its value [18].
- Equivalent classes. Equivalent classes provide both sufficient and necessary axiom assertions to define an ontology class.
- Superclasses. Superclass axioms assert the parents of an ontology class.

- In Ontorat, the above ontology axioms are formatted using the Manchester OWL Syntax, a logical syntax designed for writing OWL class expressions (http://www.w3.org/TR/owl2-manchester-syntax/) [17]. An internally designed code is used to represent different columns (*i.e.*, variables). Specifically, we use {$columnA} to represent the first column (or column A), and use {$columnB} to represent column B, etc. Each column represents a variable that will be used to define an ontology class.
- In addition, the URIs of terms, including many commonly used properties (*e.g.*, rdfs:label) shown in the axiom settings (Figure 3 (7)), need to be specified since the Ontorat program cannot know they are ontology terms unless their URIs are provided.

In addition, Ontorat requests two other types of inputs before execution.•Operation type, Figure 3 (3):

- Ontorat supports two kinds of operations based on purposes: (1) generation of new ontology classes with axioms, and (2) modification of existing ontology classes with adding new axioms. An Ontorat user is requested to specify the purpose of an Ontorat operation.
- Inputs for assigning unique URIs to newly generated terms:
- When Ontorat generates new classes, unique URIs will be assigned to newly generated terms. To achieve this task, the following information is needed:

- Target ontology, Figure 3 (1):
  A user has an option to provide a target ontology to ensure that unique ontology IDs will be assigned to newly generated ontology terms. Ontorat currently does not retrieve the information of ontology from existing ontology RDF triple store. To provide a target ontology, an Ontorat user can either upload the target ontology from a local computer or provide the URL of the target ontology.
- Start portion of term URI, Figure 3 (8):
  The start portion of term URI used for newly added terms need to be specified. For example, the string “http://purl.obolibrary.org/obo/” is used as the start portion of a URI of a term in an OBO Foundry ontology.
- Information of auto-generated term ID, Figure 3 (9):
  Three data items are needed: prefix, number of digits, and the start ID number. For example, the Vaccine Ontology (VO) terms have the prefix of “VO_” that is followed by 7 digits. We can manually specify the start ID from “1” or from another number (*e.g.*, “10000”). This feature has a pitfall since the incrementally assigned IDs from the start ID may duplicate existing IDs in the target ontology. To avoid this potential conflict, users may upload the target ontology as described above. With the target ontology provided, Ontorat will ensure the automatic generation of non-replicated IDs.

After the above information is provided manually, Ontorat can generate an input setting text file for later reuse (Figure 3), which is an important feature of Ontorat.

### Ontorat outputs

Based on a user’s request, the Ontorat can generate two kinds of outputs: an OWL file converted from a spreadsheet data file based on axiom settings, and an input setting file described above.

The Ontorat output OWL file can be visualized using OWL ontology editors such as Protégé (http://protege.stanford.edu/). The output OWL file can be imported to a target ontology (*e.g.*, VO) using the OWL import function or merged to enrich the target ontology.

It is noted that a Manchester syntax file is generated internally as an intermediate file which is used as the input to generate a final OWL output file. When an error occurs in translating the Manchester syntax to OWL format, Ontorat will be able to provide the intermediate Manchester syntax file for debugging.

### Availability

The Ontorat program is freely available on the website: http://ontorat.hegroup.org/. The source code of the Ontorat software is released and available for downloading on Github: https://github.com/ontoden/ontorat. The source code is open source with the license of Apache License 2.0.

## Features and usage

As described above, the Ontorat web application supports two operations: generation of new ontology classes with axioms, and adding new axioms to existing ontology classes. Three types of axiom assertions (for asserting annotations, equivalent classes, and superclasses) are allowed in Ontorat. In this section, we will use two specific examples to demonstrate how Ontorat supports the above features, briefly summarize other use cases, and then describe the Ontorat collection of different design patterns, templates, and examples.

### Illustration of Ontorat features using CLO and Biobank use cases

Cell lines are routinely used in various biological and biomedical studies such as analysis of cell signalling pathway studies and host-pathogen interactions [19, 20]. The Cell Line Ontology (CLO) is a community-based ontology that has logically represented over 38,000 cell line cells [16]. For this Ontorat case study, an Excel file containing information of over 1,000 cell line cells, which was obtained from the Cell Bank of RIKEN BioResource Center (BRC) in Japan, was used as input to add these cell line cell terms and their annotations into CLO [16].

**Figure 4:**
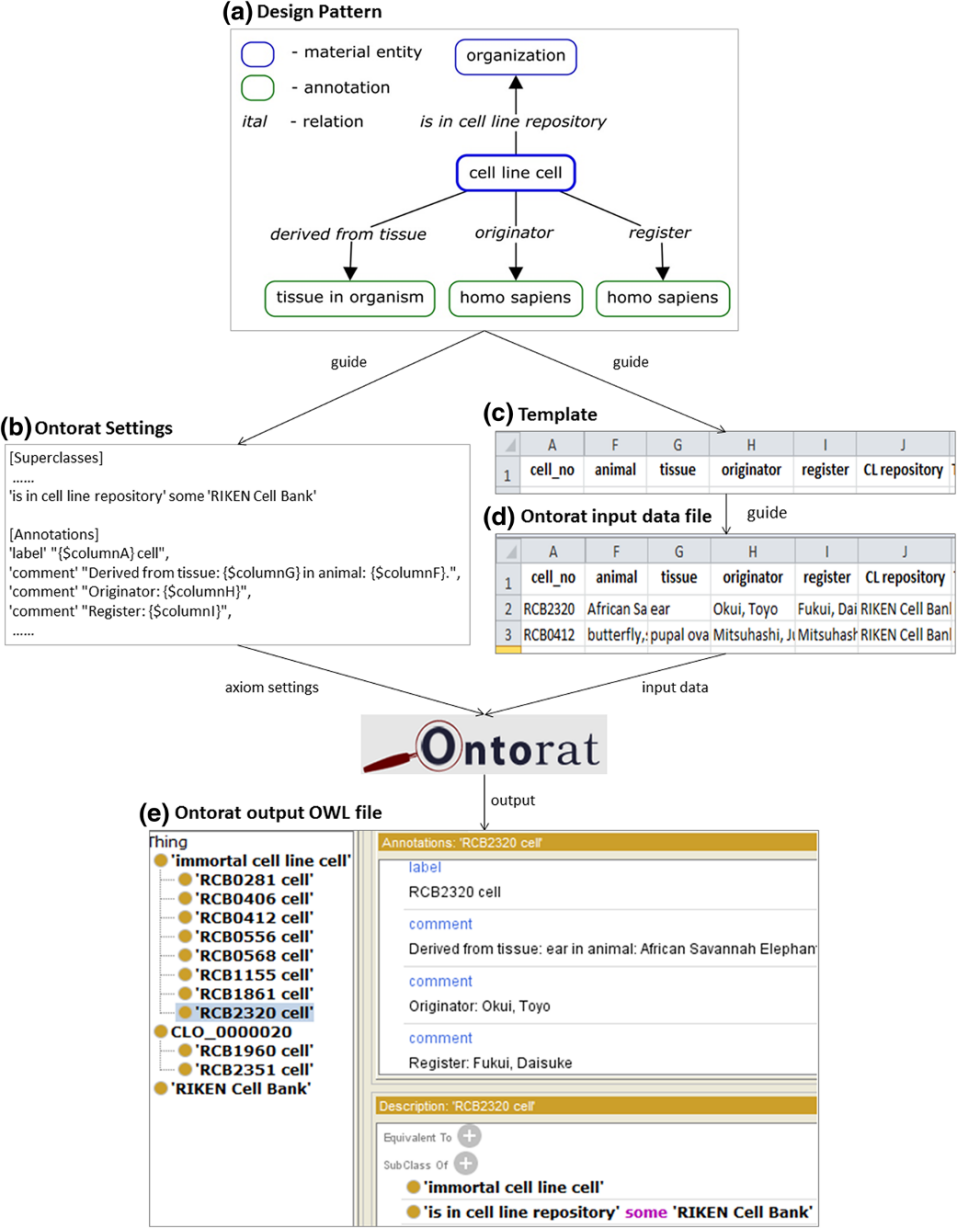
**Demonstration of an Ontorat use case for ontology enrichment.** This use case aimed to enrich the Cell Line Ontology (CLO) with new over 1,000 cell line celles collected in Japan RIKEN Cell Bank. First the ODP was identified to define these cell line cells **(a)**. As guided by the ODP, a list of Ontorat settings was generated to specify axiom expressions with possible variables of terms and annotations **(b)**. The template file **(c)** was also generated and used to fill specific contents **(d)**. Finally Ontorat generated an OWL format output file containing newly created ontology terms together with their annotations. The output could be displayed using the Protégé-OWL editor **(e)**. It is noted that only parts of Ontorat settings and input data file are shown here. The full version of the files is available on: http://ontorat.hegroup.org/designtemplates/cellline/clo-celllinecell.php.

Figure 4 demonstrates an Ontorat example based on the general strategy shown in Figure 1. Figure 4a shows the design pattern used to define cell line cells obtained from the RIKEN BioResource Center. Based on the design pattern, the following elements (terms or annotations) are needed to define a cell line cell: (i) Cell line resource (*e.g*. Japan RIKEN Cell Bank); (ii) Tissue in an organism that a cell line cell is derived from; (iii) Person(s) who registered the cell line (register); and (iv) Persons who developed or maintained the cell line (originator).

As described in the Implementation section, different assertion axioms were generated to represent the relations of terms or annotations to targeted terms (*e.g.* cell line cells) (Figure 4b). For example, cell line resource is represented as a superclass axiom expressed as follows:

‘is in cell line repository‘ some ’RIKEN Cell Bank’

This axiom specifies that the newly generated cell line cell is in the RIKEN cell line repository. To ensure that Ontorat correctly interpreted the axiom, the term URIs for both ‘is in cell line repository’ and ‘RIKEN Cell Bank’ should be specified in the web form as indicated in Figure 3(7). With these specifications, Ontorat will be able to translate the axiom into an OWL expression. The annotations of the term, such as label, are represented as annotation axioms, as demonstrated below (lower part of Figure 4b):

‘label’ “{$columnA} cell”

This axiom represents that the label of the newly added cell line cell term is defined as the string shown in the column A (represented by {$columnA}) of the input data file followed by the word “cell”. The input template file (Figure 4c) was populated with information for a specific cell line cell per row (Figure 4d). The string in the column A of the first row is “RCB2320”. Based on the above axiom setting, the label of the first cell line cell term is “RCB2320 cell” (Figure 4e).

Using the same approach, Ontorat has added the information of derived tissues, originators, and registers of individual cell line cells as annotation axioms of the newly generated cell line cell terms (lower part of Figure 4b). It is noted that in this case, we have added this information as annotations of cell line cell terms. It is also possible to add the same information as superclass axioms if we wish to. For example, instead of defining the following annotation axiom:

‘comment’ “Derived from tissue: {$columnG} in animal: {$columnF}.”

We should add the following superclass axiom assertion:

‘derived from’ some ({$columnG} ‘part of’ some {$columnF})

Where column G includes tissue information and column F includes animal information. In this case, the term ‘derived from’ should be an object property. Furthermore, instead of simple strings, specific ontology term URIs representing the tissue and animal should be provided in column G and column F, respectively. Therefore, same ODP could be represented by different OWL expressions.

The detailed Ontorat ODP, template, setting file, and the example input and output files are available on the Ontorat template web page: http://ontorat.hegroup.org/designtemplates/cellline/clo-celllinecell.php.

The above CLO example involves the generation of new ontology terms and addition of logical axioms and annotation axioms at the same time using Ontorat.

Ontorat supports editing existing terms by addition of new axioms (*e.g.* annotations). For example, Ontorat was recently used to automatically add definition source and term editor annotations to over 50 ontology classes in the Biobank Ontology (https://code.google.com/p/biobank-ontology/). The Biobank Ontology is developed for representing and annotating entities related to Biobank repositories. When new terms were initially added into the ontology, definition source and term editor were not specified. To add the annotations to biobank-specific classes, the following settings were used in the Ontorat annotations input section:

‘definition editor’ “{$columnC}”,

‘definition source’ “{$columnD}”

Since the aim of this use case is to add annotations to existing ontology terms, the ‘edit existing classes …’ option was chosen in the Ontorat Purpose input (Figure 3 (2)). The Ontorat input files used to edit Biobank Ontology and the output OWL file are available on: http://ontorat.hegroup.org/designtemplates/biobank/index.php.

### Brief summary of other Ontorat use cases

In the original Ontorat software demonstration in the ICBO-2012 conference [13], Ontorat was used to add approximately 800 US-licensed animal vaccines to the Vaccine Ontology [14, 15]. VO is a community-based ontology in the domain of vaccine and vaccination. These vaccines include 303 licensed vaccines against infections of individual pathogens and 494 combination vaccines, each of which protects against infections of two or more pathogens. The data for these vaccines were originally extracted from the official USDA website and stored in the VIOLIN vaccine database (http://www.violinet.org) [21]. Corresponding to the two sets of animal vaccines based on one or more pathogens targeted by a vaccine, two Ontorat Excel template files were generated. In addition to the generation of new classes of licensed animal vaccines, Ontorat was used to add annotations using annotation properties (*e.g.*, see_Also and term definition) [13]. To achieve multiple tasks, we performed multiple Ontorat executions, each execution to achieve a specific task.

A large number of experimental assays have been used in the biological and biomedical fields. The OBI consortium has a major focus on modeling and representing these assays [9]. OBI assays were defined by several elements including: (i) assay inputs, such as materials to be evaluated and devices used; (ii) assay output that is information about some biological process or function (e.g., gene expression, DNA methylation); (iii) assay aims, such as identification of epigenetic modification, and (iv) main processes of an assay, such as immunoprecipitation and sequencing. It is often complicated to fully represent and annotate an assay term in OWL expression. To manually generate assay term with rich axioms is very time-consuming and has become a bottleneck in OBI ontology expansion. To solve this issue, Ontorat was applied.

Since the Excel template file format is generally friendly and widely used by the public, domain experts without ontology knowledge are able to add contents to the template file. In the community-based ENCODE project [22], the OBI team developed specific template files for adding assay and device terms based on ODPs. The templates were then provided to domain experts for them to submit term requests. The requested terms with rich annotations and logical axioms were then added into OBI using Ontorat, and the ontology term IDs assigned by Ontorat were provided to the end users for their usage.

Recently Ontorat has been utilized to add mouse strain terms in the Beta Cell Genomics Ontology (BCGO) [23]. Although BCGO did not define mouse strain logically, it contains rich annotations including MGI id, common name, alternative term, definition, definition source, and term editor. The Ontorat speeded up generation of these terms. Moreover, since settings and templates can be reused, it will be easy to add more mouse strain terms in the future.

In addition to the use cases described above, Ontorat has been applied to the development of the Ontology of Vaccine Adverse Events (OVAE) [24] and the Ontology of Biological and Clinical Statistics (OBCS) [25].

### Collection of design patterns and templates

Since the ontology design pattern is a reusable modeling solution for building an ontology, the Ontorat website has provided a collection of design patterns and corresponding templates for ontology developers to reuse. For each collected case, Ontorat provides an ODP diagram, an Excel template, a setting file, and an example with populated template data and output OWL file. The collection supports the development of several ontologies, including OBI, VO, CLO, and BCGO and available on: http://ontorat.hegroup.org/designtemplates.

## Discussion

Manually adding a large amount of terms or terms with rich axioms into an ontology is a big challenge and become a bottleneck of ontology development. It is time consuming and error-prone to do it manually. Many ontology terms were generated with the same ontology design patterns (ODPs). Based on ODPs and inspired by the Quick Term Template (QTT) procedure, the Ontorat web application is developed to provide a robust and scalable platform for automatically generating new ontology terms, axioms and annotations. Ontorat supports efficient ontology enrichment and expansion. The design patterns can be reused by ontology developers. The Ontorat spreadsheet templates lower the technical barriers for domain experts and data curators, so that they may contribute actively to the ontology development without knowing the specifics of OWL.

Tools with similar functions to Ontorat exist, including MappingMaster [4], Populous [26], and TermGenie (http://code.google.com/p/termgenie/). As introduced in the Background section, as a Protégé plugin, MappingMaster can only be used with old version Protégé 3.4 and has not been updated to work for commonly used Protégé 4 and 5 [4]. In addition, MappingMaster requires writing template class expression using a M2 language, a Domain Specific Language (DSL) based on the Manchester OWL syntax. The programming with the language requires a learning curve. In contrast to MappingMaster, Ontorat can build axiom expressions from a web form using the Manchester syntax. Ontorat has the capability of automatically generating annotations of ontology terms. Populous provides desktop standalone and user-friendly interface [26]. However, it needs software installation. Populous does not support the generation of term annotations. Ontorat is implemented as a user-friendly web-based application without the necessity of software download and installation. TermGenie provides a web application that creates new terms for an ontology using patterns (http://code.google.com/p/termgenie/). TermGenie has been used for the Gene Ontology (GO) and its cross products (http://go.termgenie.org/). Based on predefined patterns, TermGenie supports new ontology term generation and provides a user-friendly interface to domain experts. Compared to Ontorat, TermGenie does not allow the generation of new terms based on user-provided patterns. Ontorat provides more flexibility in allowing users to define patterns for different ontologies. TermGernie cannot be used to add new axioms to existing terms.

To ensure the generation of unique IDs for newly generated terms for a target ontology, the ontology is currently required to be loaded in Ontorat. URIGen is a Java API and web service for managing ontology URI creation (http://www.ebi.ac.uk/fgpt/sw/urigen/). URIGen also provides a REST interface that interacts with the URIGen server. It is possible to incorporate the URIGen distributed ID management functionality into Ontorat for unique ID assignment.

While Ontorat is primarily targeted for ontology developers with sufficient OWL ontology background, Ontorat provides a way to separate the duties from the ontology developers and domain experts who both participate in the development of a specific domain ontology. Ontorat separates the Manchester syntax programming from the template spreadsheet population. A domain expert who does not know programming can still work on the ontology development project by working on populating the Excel spreadsheet. For example, in the OBI Assay example described above, after receiving the Assay Excel template file, the domain experts in the ENCODE project [22] were able to independently provide definitions and other information needed to define an assay term. After obtaining the Excel file from the ENCODE group, the OBI developers were able to use Ontoat to generate new ontology terms and annotations separately. The logical axiom expressions in Ontorat use the standard and widely-used Manchester syntax, together with simple Ontorat rules for representing ontology variables. Therefore, Ontorat provides a relatively straight forward platform for ontology developers who are familiar with the Manchester syntax, which is also used in the Protégé-OWL editor.

Ontorat implements the Quick Term Template (QTT) procedure and more. An Ontorat template is equivalent to a QTT template when the template is designed for generating new ontology classes for ontology expansion. In addition to new class generation, Ontorat can also support the addition of new annotations to existing ontology classes. In the future, Ontorat will also support the generation of axioms that contain instances. Different from the QTT approach, Ontorat emphasizes the generation of machine-readable and reusable axiom setting file. The Ontorat axiom expressions use the Manchester OWL syntax and easy-to-use Ontorat syntax of variables. The Ontorat syntax provides a way to represent variables that are mapped to columns in the Excel template spreadsheet. The Ontorat generated Ontorat setting file is easily understandable and reusable.

Among software programs that support ontology development, Ontorat is complementary to OntoFox (http://ontofox.hegroup.org), another web application developed by our group with the support from the OBO Foundry community [27]. OntoFox supports the retrieval of a subset of ontology terms and axioms from existing ontologies [27]. Ontorat and OntoFox are complementary in the sense that OntoFox supports the reuse of existing ontology terms and Ontorat supports the automatic generation of new ontology terms, axioms and annotation of ontology terms. OntoFox and Ontorat have been combined in use for development of new ontologies, such as the Cell Line Ontology (CLO) [16], Vaccine Ontology [28], Ontology of Biological and Clinical Statistics (OBCS) [25], and Beta Cell Genomics Ontology (BCGO) [23]. In fact, Ontorat and OntoFox are developed using similar web-based form and setting file design. For example, the Ontorat setting file is similar in spirit to the OntoFox setting file that has been proven to be very useful for reusability. We will seek ways to better integrate these two software programs for more efficient ontology development.

Furthermore, we plan to expand the Ontorat collection of ODPs, templates, and setting files together with examples. Such a collection will support ontology design pattern reuse, standardization, and various applications. We encourage all parties to participate in contributing their domain knowledge and expertise in this collaborative movement.

Ontorat was introduced in an OBO Tutorial in the International Conference on Biomedical Ontologies (ICBO) in 2013. The tool was also demonstrated in an OBO Tutorial and an OBO Technical Workshop in ICBO-2014 (http://icbo14.com/), held at Houston, Texas, USA. Given strong community demands and support, Ontorat has provided a timely platform to support efficient ontology development and applications.

## Conclusions

Ontorat (http://ontorat.hegroup.org) is a web application that supports automatic generation of new ontology terms, term annotations, and logical axioms. Ontorat allows the storage and reuse of axiom setting files and input template files. Ontorat has also started the collection of reusable ontology design patterns and templates.

## Abbreviations

BCGO: Beta cell genomics ontology
CLO: Cell line ontology
GO: Gene ontology
ICBO: International conference on biomedical ontology
OBCS: Ontology of biological and clinical statistics
OBI: Ontology for biomedical investigations
OBO: Open biology and biomedical ontologies
OVAE: Ontology of vaccine adverse events
VO: Vaccine ontology.
